# Rottlerin-Mediated Inhibition of *Chlamydia trachomatis* Growth and Uptake of Sphingolipids Is Independent of p38-Regulated/Activated Protein Kinase (PRAK)

**DOI:** 10.1371/journal.pone.0044733

**Published:** 2012-09-07

**Authors:** Lei Lei, Zhongyu Li, Guangming Zhong

**Affiliations:** 1 Department of Microbiology and Immunology, University of Texas Health Science Center at San Antonio, San Antonio, Texas, United States of America; 2 University of South China, Hengyang, Hunan, China; University of California Merced, United States of America

## Abstract

We previously found that rottlerin, a plant-derived small molecule compound, profoundly inhibited *Chlamydia trachomatis* growth and blocked sphingolipid trafficking from host cell Golgi into chlamydial inclusions. Since the p38-regulated/activated protein kinase (PRAK) is a known target of rottlerin and is activated in *Chlamydia trachomatis*-infected cells, we investigated the potential role of this kinase in rottlerin-mediated anti-chlamydial activity. However, we found that a PRAK-specific inhibitor failed to inhibit chlamydial growth, suggesting that the kinase activity of PRAK may not be required for chlamydial intracellular replication. This conclusion was supported by the observation that chlamydial organisms replicated equally well in mouse embryonic fibroblast cells with or without PRAK. Moreover, neither the PRAK inhibitor nor PRAK deficiency altered host sphingolipid trafficking into chlamydial inclusions. Finally, rottlerin maintained its anti-chlamydial activity in PRAK-deficient cells. Together, these observations have demonstrated that PRAK is not required for either the rottlerin-mediated anti-chlamydial activity or rottlerin inhibition of sphingolipid trafficking, suggesting that rottlerin may achieve its inhibitory role by targeting other host factors.

## Introduction

Urogenital tract infection with *Chlamydia trachomatis* is the number one cause of sexually transmitted bacterial diseases worldwide [1], [2]. Although the precise pathogenic mechanisms on how C. *trachomatis* infection causes diseases remains unclear, it is thought that the obligate intracellular growth and cell to cell spreading may significantly contribute to the C. *trachomatis* pathogenicity. The infectious elementary bodies (EB) of C. *trachomatis* invade the epithelial cells of urogenital tract via endocytosis. An intracellular EB then differentiates into a non-infectious but metabolically active reticulate body (RB) for biosynthesis and replication. The progeny RBs finally differentiate back into EBs for spreading to new cells. The whole process is restricted within a cytoplasmic vacuole termed inclusion. Thus, nutrients and energy must be transported into the inclusions to support chlamydial growth. For example, sphingomyelin synthesized from fluorochorme-labeled ceramide in the Golgi apparatus was detected in the chlamydial inclusions [3], [4], [5].

Despite the serious health problem caused by *C. trachomatis* infection, many infected individuals are undiagnosed and untreated due to lack of obvious symptoms. Thus, searching for non-antibiotics topical reagents that can prevent *C. trachomatis* transmission is necessary. Previously, we reported that rottlerin profoundly inhibited chlamydial intracellular growth, which correlated with blockade of sphingolipid trafficking from the host cell Golgi apparatus into chlamydial inclusions [5]. The rottlerin anti-chlamydial activity appeared to depend on its ability to target host factors since the rottlerin blockade of chlamydial growth was strongest when rottlerin was used to treat host cells prior to or immediately after infection but significantly weakened as the treatment delayed 16h after infection [5]. Indeed, rottlerin is known to inhibit various host kinases including PKCδ [6], CaM Kinase III [7] and p38-regulated/activated kinase(PRAK) [8]. Since PRAK is activated during *C. trachomatis* infection, we tested whether PRAK plays a role in rottlerin’s anti-chlamydial activity in the current study.

PRAK, also known as MAPK-activated protein kinase 5 (MK5), is phosphorylated and activated by p38^MAPK^ [9] and ERK2 [10]. PRAK has been shown to phosphorylate several substrates including HSP27, p53, FoxO3 and Rheb [11]. The biological role of PRAK remains incompletely understood. PRAK has been shown to complex with a tumor suppressor [12], [13] and regulate actin polymerization and cell motility [14], [15]. During chlamydial infection, MAP kinase pathways are activated, which has been shown to contribute to chlamydial uptake of host glycerophospholipid [16]. In the current study, we found that PRAK was activated during chlamydial infection and rottlerin both inhibited the PRAK activity and blocked chlamydial intracellular growth, suggesting that PRAK might play an important role in rotterlin anti-chlamydial activity. However, evidence presented in the current manuscript has demonstrated that rottlerin inhibition of both chlamydial growth and acquisition of host sphingolipids is independent of PRAK.

**Figure 1:**
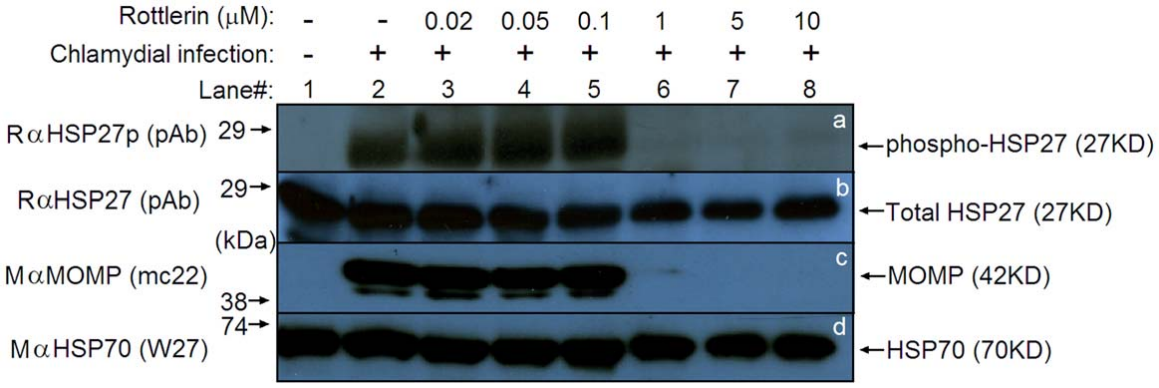
Rottlerin inhibits both HSP27 phosphorylation and chlamydial growth. HeLa monolayer cells grown in 24 well plates were infected with or without *Chlamydia trachomatis* infection (MOI = 1) and 16 h post infection, the cultures were treated with or without rottlerin at different concentrations as indicated on top of the figure. The culture samples were harvested 48 h post infection and analyzed on Western blot for detection of phosphorylated HSP27 (phospho-HSP27, panel a), total HSP27 (b), chlamydial major outer membrane protein (MOMP, c) and host HSP70 as a loading control (d) with the corresponding antibodies as listed in the left of the figure. Note that neither phospho-HSP27 nor MOMP was detectable in the infected cultures treated with 1 µM or higher concentrations of rottlerin (lanes 6–8).

## Materials and Methods

### 1. Chlamydial Infection

The *C. trachomatis* L2/LGV-434/Bu organisms used in the current study were propagated, purified, aliquoted and stored as described previously [17], [18]. HeLa cells (human cervical carcinoma epithelial cells, ATCC cat# CCL2), wild type or PRAK deficient mouse embryo fibroblasts (MEF) (kindly provided by Dr. Jiahuai Han, Scripps Research Institute, La Jolla, CA; ref: 12) were grown in either 24-well plates with coverslips or tissue culture flasks containing DMEM (GIBCO BRL, Rockville, MD) with 10% fetal calf serum (GIBCO BRL) at 37°C in an incubator supplied with 5% CO2. These cells were inoculated with chlamydial organisms at the appropriate MOIs as indicated in individual experiments. The infected cultures were processed at various time points after infection for either immunofluorescence assays or Western blot analyses as described below.

### 2. Western Blot

Western blot assay was carried out as described elsewhere [19]. Briefly, HeLa cells were infected with *C. trachomatis* for 16 h or various periods of time as indicated in individual experiments and treated with the corresponding inhibitors including rottlerin [5,7-dihydro-2,2-dimethyl-6-(2,4,6-trihydroxy-3-methyl-5-acetylbenzyl)-8-cinnamoyl-1,2-chromene, cat#R-1120, A.G scientific Inc, San Diego, CA] and EGCG (epigallocatechin 3-gallate, cat#E4143, Sigma, St. Louis, MO). At 48 h post infection, cell samples were harvested and subjected to electrophoresis in a SDS polyacrylamide gel. The resolved protein bands were transferred to nitrocellulose membranes for antibody detection. The following primary antibodies were used: rabbit polyclonal antibody (pAb) against HSP27 (cat#06–478, upstate biotechnology, Billerica, MA), rabbit pAb against phosphorylated HSP27 at serine 78 (p-HSP27, cat#sc-16568-R, Santa Cruz Biotechnology, Santa Cruz, CA), mouse monoclonal antibody (mAb) MC22 against chlamydial major outer membrane protein (MOMP; ref: [20] and mAb W27 against host cell HSP70 (cat#Sc-24, Santa Cruz Biotechnology). The primary antibody binding was probed with an HRP (horse radish peroxidase)-conjugated goat anti-mouse IgG or goat anti-rabbit IgG secondary antibody (Jackson ImmunoResearch, Inc, West Grove, PA) and visualized with an enhanced chemiluminescence (ECL) kit (Santa Cruz Biotech).

**Figure 2:**
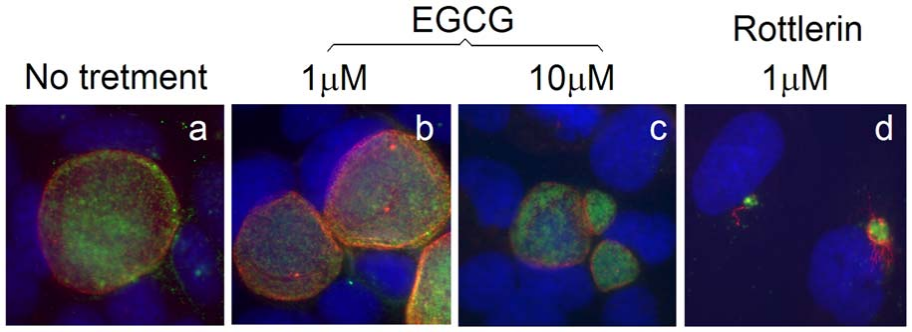
EGCG fails to inhibit chlamydial growth. HeLa cells infected with *C. trachomatis* (MOI = 0.5) were treated with EGCG (1 µM, panel b & 10 µM, c) or rottlerin (1 µM, d) 16 h post infection. The cultures were processed 44 h post infection for immuno-labeling with a rabbit antibody for labeling the *C. trachomatis* organisms (green) and a mouse monoclonal antibody (clone BB2) for inclusion membrane protein IncA (red). Note that EGCG at 1 µM failed to inhibit inclusion expansion and even at 10 µM only slightly reduced inclusion size while rottlerin at 1 µM completely blocked inclusion expansion.

**Figure 3:**
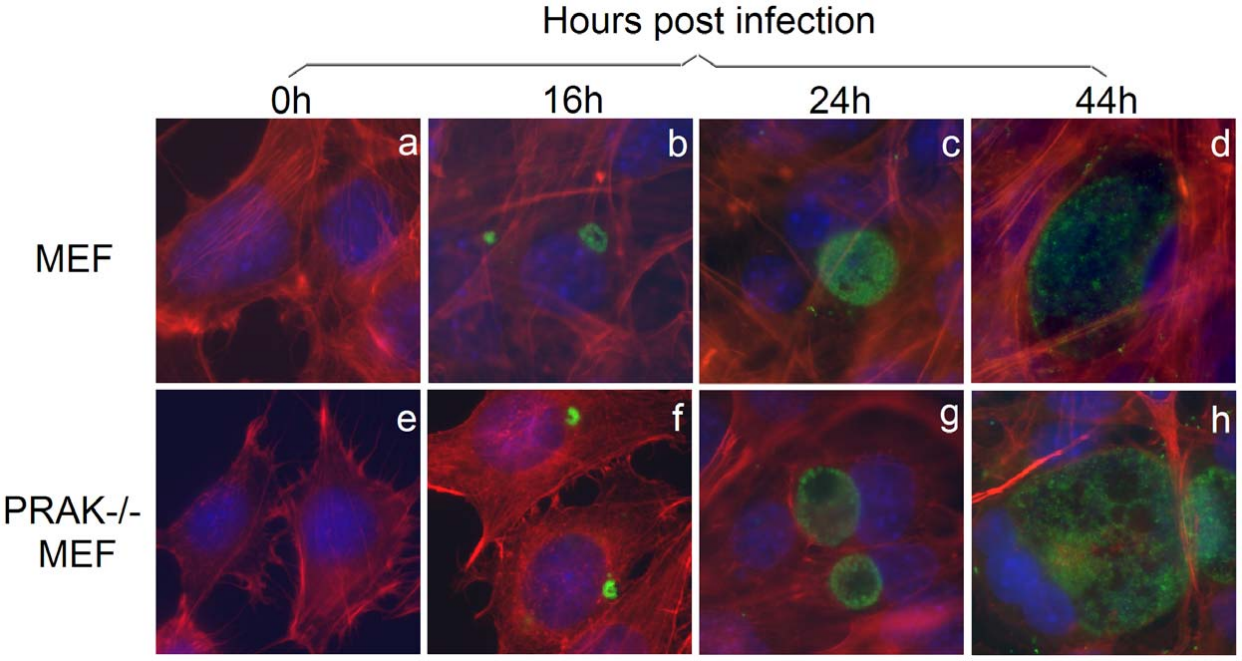
Replication of *C. trachomatis* organisms in PRAK-deficient mouse embryo fibroblast cells. Mouse embryo fibroblast cells (MEF) without (panels a–d) or with (e–h) PRAK deficiency (PRAK−/−) were infected with (b–d & f–h) or without (a & e) *C. trachomatis* (MOI = 0.5) for various periods of time as indicated on top of the figure. The cultures were processed for immunofluorescence assay with a rabbit antibody for visualizing chlamydial organisms (green), Alexa-Fluor 568 Phalloidin for host cell F-actin (red) and Hoechst dye for DNA (blue). Note that the inclusion sizes were similar in MEF with or without PRAK deficiency.

**Figure 4:**
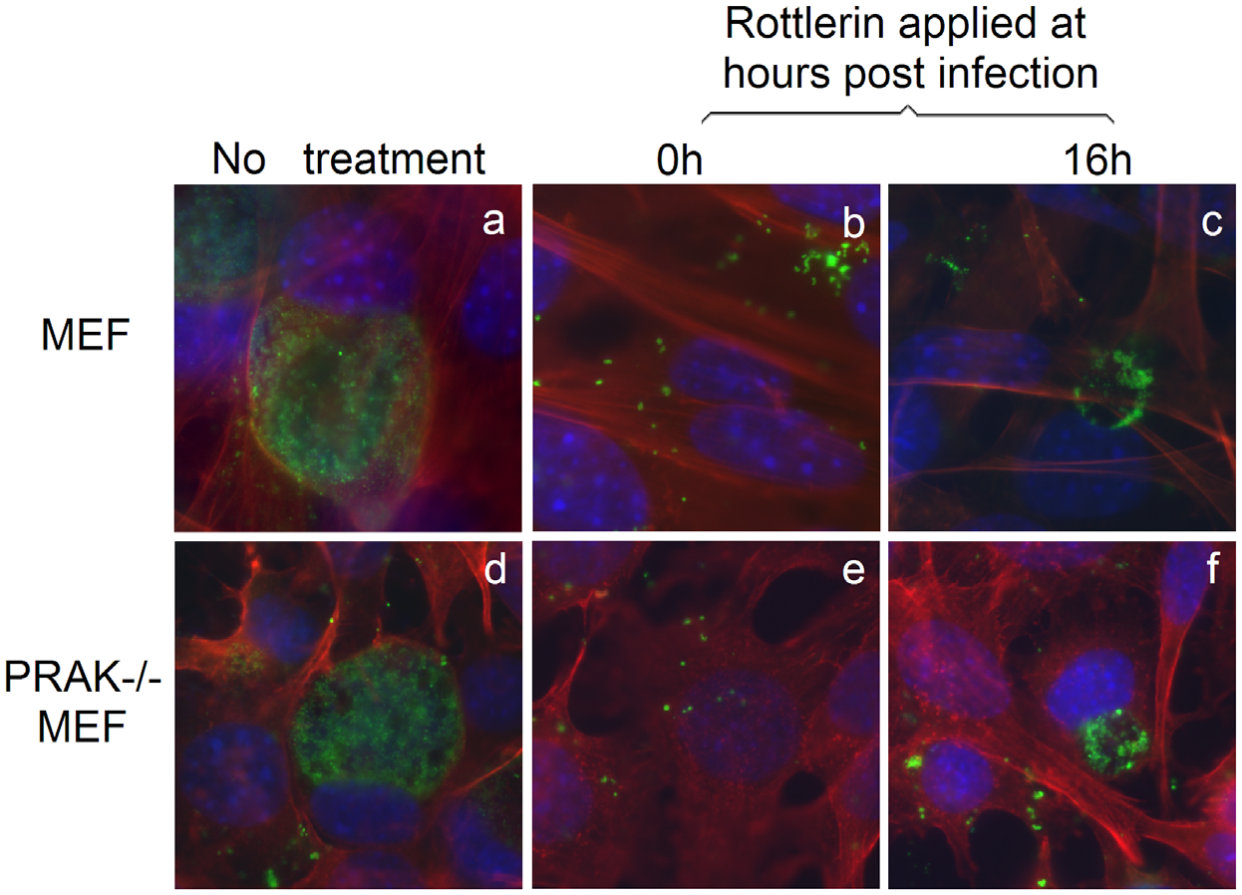
Rottlerin inhibits chlamydial growth in the absence of PRAK. MEF without (panels a–c) or with (d–f) PRAK deficiency (PRAK−/−) were infected with *C. trachomatis* (MOI = 0.5) and at oh (b & e) or 16 h (c & f) post infection, parallel cultures were treated with (b, c, e & f) or without (a & d) rottlerin at 1 µM. The cultures were processed 44 h post infection for immunofluorescence assay as described in Fig. 3 legend. Note that rottlerin inhibited chlamydial growth in both wild type and PRAK-deficient MEF cells.

### 3. Sphingomyelin Accumulation Assay

The sphingomyelin accumulation assay was carried out as described previously [3], [5]. HeLa or wild type or PRAK-deficient MEF cells were infected with *C. trachomatis* for 16 h and then treated with the inhibitors as indicated in individual experiments for an additional 8 h. At 24 h post infection, live cells were incubated for 30 minutes in serum-free MEM containing 1 µM BODIPY-FL-C5-ceramide (cat#D3521, Invitrogen, Grand Island, NY) complexed with 0.034% defatted bovine serum albumin (dfBSA) (Sigma) in MEM at 4°C in the dark. Cells were washed 3 times with Hank’s balanced salt solution and back exchanged with MEM/0.34% dfBSA for 1 hour. After wash, cells were immediately visualized by fluorescence microscope.

**Figure 5:**
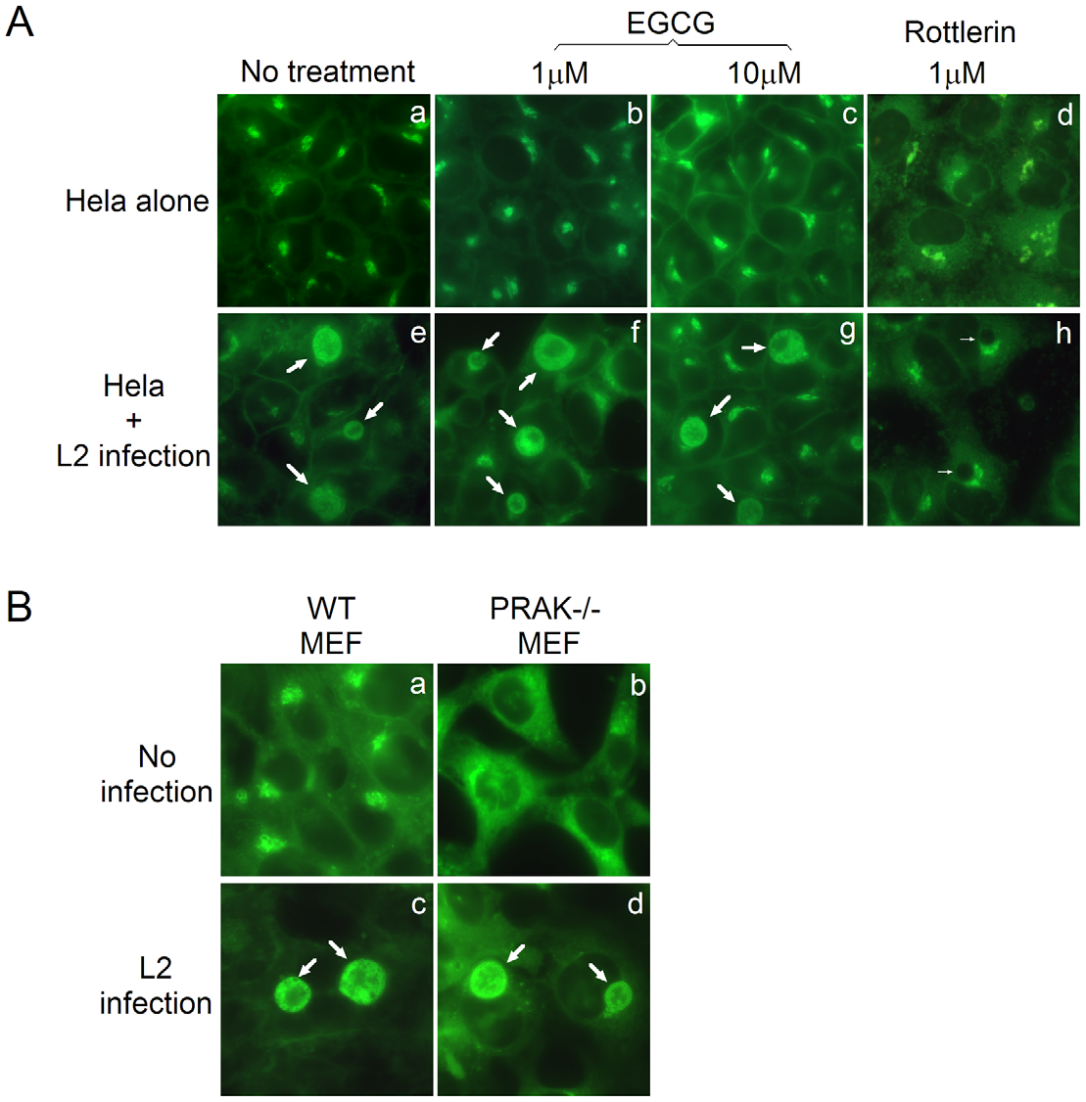
*Chlamydia trachomatis* acquisition of host sphingomyelin is independent of PRAK. (A) HeLa cells with (panels e–h) or without (a–d) *C. trachomatis* infection (MOI = 0.5) were treated without (a & e) or with EGCG (1 µM, b & f; 10 µM, c & g) or rottlerin (1 µM, d & h) 16 h post infection. Eight hours later, the cultures were subjected to BODIPY-FL-C5-ceremide labeling and visualized under a fluorescence microscope. Note that EGCG failed to block the accumulation of BODIPY-FL-sphingomyelin in the chlamydial inclusions (panel f & g) while rottlerin did (h). (B) MEF without (panels a & c) or with (b & d) PRAK deficiency (PRAK−/−) were infected with *C. trachomatis* (MOI = 0.5) and 24 h post infection, the cultures were labeled with BODIPY-FL-C5-ceremide and observed as described above. Note that *C. trachomatis* organisms can take up BODIPY-FL-sphingomyelin from MEF cells with or without PRAK. The thick arrows point to chlamydial inclusions with while thin arrows point to the inclusions without the fluorescent sphingomyelin.

### 4. Immunofluorescence Assay

The immunofluorescence assay was carried out as described previously [21]. Briefly, HeLa or MEF cells with or without chlamydial infection grown on coverslips were fixed with 2% paraformaldehyde for 30 min at room temperature, followed by permeabilization with 2% saponin (both from Sigma) for an additional 30 min. After washing and blocking, the cell samples were subjected to antibody and chemical staining. A rabbit anti-chlamydial organism antibody (R1L2, raised with *C. trachomatis* L2 organisms, unpublished data) plus a goat anti-rabbit IgG secondary antibody conjugated with Cy2 (green, Jackson ImmunoResearch Laboratories, Inc) was used to visualize chlamydial organisms. Hoechst (blue, Sigma) was used to visualize DNA. In some experiments, the host cell cytoplasm was visualized by labeling the F-actin with Alexa-Fluor 568 Phalloidin (red, cat#A12380, Invitrogen).

## Results

### 1. PRAK is Activated during Chlamydial Infection and Rottlerin Inhibits both PRAK Activity and Chlamydial Intracellular Growth

To understand the mechanism of the rotterlin anti-chlamydial activity, we monitored the activity of the p38-regulated/activated protein kinase (PRAK), a known target of rotterlin, in HeLa cells with or without chlamydial infection and in the presence or absence of rotterlin by detecting the phosphorylation of host cell HSP27, a known substrate of PRAK (Fig. 1). The phosphorylation of serine residue at position 78 of HSP27 has been extensively used to indicate PRAK kinase activity [9]. A significant level of phosphorylated HSP27 was detected in Chlamydia-infected cells (as indicated by the presence of chlamydial major outer membrane protein, MOMP) but not in normal HeLa cells 48 hours after infection, demonstrating that chlamydial infection significantly activated PRAK. However, when rottlerin was added 16 hours after infection at a concentration of 1 µM, the phosphorylated HSP27 and the protein MOMP were no longer detectable although the total protein levels of both host HSP27 and HSP70 were similar between cultures with or without rottlerin treatment. These observations demonstrated that both HSP27 phosphorylation and chlamydial growth were inhibited by rotterlin, correlating the rottlerin anti-chlamydial activity with its inhibition of PRAK.

### 2. PRAK is Required for Neither Chlamydial Intracellular Growth Nor the Rottlerin Anti-Chlamydia Activity

We next tested whether the PRAK activity is required for chlamydial growth. *C. trachomatis*-infected cultures with or without treatment with either EGCG, a known inhibitor for PRAK with an IC_50_ = 1 µM [22], or rotterlin were monitored for inclusion sizes under an immunofluorescence microscope (Fig. 2). We found that the chlamydial inclusions were as big as those in untreated cultures when EGCG was used at 1 µM. Even when EGCG was used at the concentration of 10 µM, chlamydial inclusion sizes were only slightly reduced. However, treatment with rottlerin at 1 µM almost completely blocked chlamydial intracellular growth. These observations suggest that PRAK may not be required for chlamydial growth. This conclusion is validated by the result that *C. trachomatis* organisms replicated equally well in both wild type and PRAK-deficient MEF cells (Fig. 3). Thus, it is unlikely that the rotterlin anti-chlamydial activity requires PRAK. We indeed found that rottlerin efficiently inhibited chlamydial growth in both wild type and PRAK-deficient cells (Fig. 4).

### 3. PRAK is not Required for Sphingolipid Trafficking into Chlamydial Inclusions

We also examined the role of PRAK in host sphingolipid trafficking into chlamydial inclusions. The PRAK inhibitor EGCG even at 10 µM failed to affect sphingolipid accumulation inside chlamydial inclusions while rottlerin at 1 µM completely blocked sphingolipid trafficking into chlamydial inclusions although neither inhibitors significantly alter the Golgi accumulation of sphingolipids (Fig. 5A), suggesting that chlamydial uptake of sphingolipids from host cells does not require PRAK. This conclusion is further supported by the observation that host sphingolipids accumulated in chlamydial inclusions in MEF cells with or without PRAK gene deficiency (Fig. 5B).

## Discussion

Rottlerin is known to inhibit various host kinases including PRAK. Since PRAK is activated during *C. trachomatis* infection and rottlerin inhibited both the PRAK activity and chlamydial intracellular growth, we evaluated the role of PRAK in rottlerin anti-chlamydial activity. However, our experimental results have led us to conclude that PRAK is not required for either the rottlerin inhibition of chlamydial growth or sphingolipid uptake. First, we have shown that a PRAK-specific inhibitor fails to inhibit chlamydial growth; Second, chlamydial organisms replicate equally well in mouse embryonic fibroblast cells with or without PRAK; Third, the PRAK inhibitor nor PRAK deficiency can alter host sphingolipid trafficking into chlamydial inclusions; Finally, rottlerin can maintain its anti-chlamydial activity in PRAK-deficient cells. These observations together suggest that rottlerin may achieve anti-chlamydial activity by targeting other host factors.

The question is what host factors might contribute to the rottlerin anti-chlamydial activity. Rottlerin can inhibit host PKCδ [6], CaM Kinase III [7] and p38-regulated/activated kinase (PRAK) [8]. However, PRAK alone is not required for the rottlerin anti-chlamydial activity. Thus, it will be worth testing the roles of PKCδ and CaM Kinase III in rottlerin-mediated inhibition of chlamydial growth. It is also possible that a combined inhibition of all 3 kinases by rottlerin may be responsible for the profound anti-chlamydial activity. Alternatively, rottlerin may have unknown targets that may contribute to rottlerin’s ability to inhibit chlamydial intracellular growth. For example, rottlerin can act as a mitochondrial uncoupler [23], induce apoptosis of human tumor cell lines, and potentiate chemotherapy-induced cytotoxicity [24], none of which can be explained by the rottlerin inhibition of the 3 known kinases. On the other hand, it has been shown that chlamydial organisms have evolved multiple strategies for manipulating host pathways, some of which may be redundant, in order to ensure adequate supply of host nutrients and energy to support chlamydial biosynthesis and replication inside the inclusions. For example, Chlamydia activates Raf/EMK/ERK/cPLA2 pathways to facilitate chlamydial uptake of host glycerophospholipid [16]. Chlamydia can also co-opts GBF1 and CERT to acquire host sphingomyelin [25]. Can rottlerin affect these pathways? Testing these and other hypotheses is underway.
